# Dentists' Attitudes in Jordan towards the Shortened Dental Arch Concept: A Cross-Sectional Study

**DOI:** 10.1155/2019/4163851

**Published:** 2019-12-04

**Authors:** Motasum Abu-Awwad, Rula Amarin, Farah Khouli, Sirin Shaban, Sandra AlTarawneh

**Affiliations:** ^1^University of Jordan, School of Dentistry, Amman, Jordan; ^2^University of Maryland, School of Dentistry, Baltimore, MD, USA

## Abstract

**Aims:**

To assess the awareness, knowledge, attitudes, and application of the shortened dental arch (SDA) concept by dentists in Jordan.

**Materials and Methods:**

In this cross-sectional survey, a questionnaire was disseminated to a random sample of 150 dentists working in private practices, university hospitals, or governmental institutes. The chi-square (*X*^*2*^) test was used to examine associations.

**Results:**

One hundred and six dentists responded (70.7% response rate). Fifty-five were females. 82.1% were aware of the SDA concept. The fewer the years of experience, the more likely the dentists were aware of the SDA concept (*X*^*2*^, *P*=0.024) and the more likely they learned about it through undergraduate education (*X*^*2*^, *P* < 0.001). In a hypothetical clinical situation of a patient >50 years of age with missing molar teeth, 45.3% agreed that the molars should be replaced, while 54.7% did not agree. Improving mastication was the main reason for agreeing (81.6%), while having reduced functional benefit was the main reason for disagreeing (64.9%). The treatment modality most commonly recommended was implants fixed partial dentures (84.9%). Of those aware of SDA, 67.8% agreed it could have a useful place in treatment planning within Jordan. Cost reduction for patients was the main reason for this answer, as reported by 51% of those who agreed. 26.4% did not apply SDA for any of their patients, while 50.6% applied it for <10% of their patients.

**Conclusion:**

The majority of the dentists was aware of the SDA concept and had a positive attitude towards it; however, few of them applied it in their practice.

## 1. Introduction

A paradigm shift has occurred in dentistry in the last decades. The conventional morphologically-based approach, which emphasized replacing all missing teeth to maintain complete dental arches, is being replaced with a more conservative problem-oriented approach [1–5]. Research has demonstrated that dentitions consisting of anterior and premolar teeth were able to meet oral functional and esthetic demands in the middle-aged and elderly patients and that replacement of molar teeth was not always necessary [6, 7]. This treatment approach was referred to as the shortened dental arch (SDA) concept [8–10].

Removable partial dentures (RPDs), implant supported fixed partial dentures (IFPDs), and cantilevered fixed partial dentures (CFPDs) are all examples of treatment modalities which could be used to replace missing posterior teeth [3]. However, these treatment modalities are not without biological and financial costs [4, 5]. One of the main advantages of the SDA concept is its cost effectiveness in reducing the need for treatment and reducing the burden of maintenance [8].

A few reports are available in the literature describing the attitudes among dentists towards the SDA concept [11–18]. These studies reflected a wide variation between dentists in different countries in terms of their knowledge and application of the SDA concept. It is apparent from these studies that the SDA is still neither universally accepted nor is it being commonly practiced. To the best of the author's knowledge, a study about the attitudes of dentists towards the SDA concept in Jordan has not been conducted. Medical and dental care in Jordan, like many other developing countries, takes up a large share of public spending. Implementing cost-effective treatment approaches would be beneficial for the economy [19, 20].

Therefore, the aims of the current study were to (1) evaluate dentists' awareness and knowledge of the SDA concept in Jordan and (2) assess dentists' attitudes towards the SDA concept and its application in clinical practice.

## 2. Materials and Methods

The present study was conducted in full accordance with the World Medical Declaration of Helsinki and conformed to the STROBE statement for observational studies. The study protocol was granted an ethical approval with a favorable opinion by the Deanship of Academic Research at the institute at which this study was conducted.

A self-designed questionnaire about the SDA concept along with a consent form was disseminated through email to a random sample of 150 dentists in Jordan. The sample included dentists who were working in private practice, university hospital, or a governmental institute. The sample was randomly selected from a database of all the registered dentists in the country available from the Jordanian Dental Association. The database included 3555 dentists (accessed in May 2019). The sample size was calculated using “Sample Size Calculator” available freely online (https://www.abs.gov.au/websitedbs/d3310114.nsf/home/sample+size+calculator). Using a confidence level of 95% and confidence interval of 10%, a sample size of 94 was calculated. A larger sample was invited to account for a reduced response rate. A computer-generated random table was used to select a random sample. Only dentists who signed the consent form and underwent their undergraduate education in Jordan were included in this study.

The questionnaire was derived from previous similar studies [11, 18] and was piloted and validated across a group of 10 dentists before the initiation of the study. The questionnaire contained eleven questions which were distributed across three sections. The first section gathered demographic information (gender, years of clinical experience, level of education, and work environment whether private practice, university hospital, or a governmental institute).

The second section targeted dentists' awareness and knowledge of the SDA concept. The questions inquired whether the dentists were aware of the SDA concept and where they learned about it. Subsequently, a hypothetical clinical situation of a healthy patient over 50 years of age with missing molar teeth was presented. The patient was described to have Angle's class I occlusion with no evidence of parafunctional habits, pre-existing temporomandibular dysfunction, or any marked reduction in alveolar bone support. The dentists were asked whether they agreed that the molar teeth should be replaced for such a patient or not. This was followed by questions regarding the reasons behind their answer and the most commonly recommended treatment modality for managing such clinical situations in their daily practice.

The third section targeted dentists' attitudes towards the SDA concept, the reason behind it, and its application in their clinical practice. Only those who were aware of the SDA concept were asked to fill this section. The dentists were asked whether they believed the SDA concept could have a useful place in treatment planning in Jordan and to clarify the reason behind their answer. Lastly, they were asked about the percentage of patients in which they have applied the SDA concept as a treatment option.

Data were analyzed using the statistical package SPSS [21]. The results were summarized in tables, and associations were examined using the chi-square (*X*^*2*^) test and Fisher's exact test.

## 3. Results

### 3.1. Demographics

106 out of 150 clinicians completed the questionnaire, which accounted to a response rate of 70.7%. The distribution of the sample according to gender, work environment, level of education, and years of clinical experience was summarized in Table 1.

### 3.2. Awareness and Knowledge of the SDA Concept

Eighty-seven of the dentists (82.1%) reported they were aware of the SDA concept. There was no association between awareness of the SDA concept and the different work environments, *X*^*2*^(2, *N*: 106) = 0.025, *P*=0.988. The percentage of awareness was nearly similar across the three different work environments (82.2% private practice, 81.2% university hospital, and 82.8% governmental institute). Similarly, there was no association between awarness of the SDA concept and the level of education, Fisher's exact test (2, *N*: 106) = 1.093, *P*=0.653.

Table 2 reported the distribution of dentists who were aware/unaware of the SDA concept and where they learned about it, according to the years of clinical experience. A statistically significant association between the years of clinical experience and being aware of the SDA concept was detected, *X*^*2*^(3, *N*: 106) = 9.444, *P*=0.024. A statistically significant association was also detected between the years of clinical experience and learning about the SDA concept during undergraduate education, *X*^*2*^(3, *N*: 90) = 18.595, *P* < 0.001.

When asked about the hypothetical clinical situation of a patient above 50 years of age with missing molar teeth, 48 (45.3%) agreed that the molars should be replaced, while 58 (54.7%) did not agree. A chi-square test revealed no statistically significant association between dentists' awareness of the SDA concept and whether they agreed with the need to replace the missing molar teeth or not, *X*^*2*^(1, *N*: 106) = 2.985, *P*=0.084 (Table 3).

Dentists who agreed that the molars should be replaced reported the following reasons for their decision: improving mastication (81.6%), restoring posterior support (63.3%), preventing anterior tooth wear (53.1%), maintaining the health of the TMJs (51%), satisfying patients' desires (42.9%), avoiding the risk of tooth migration (22.4%), avoiding creation of speech problems, (10.2%) and improving esthetics (8.2%).

Dentists who did not agree that the molars should be replaced reported the following reasons for their decision: having reduced functional benefit (64.9%), reducing treatment cost (54.4%), having reduced adverse esthetic effect (49.1%), simplifying oral hygiene (38.6%), and being more conservative (31.6%).

When the dentists were asked to assume that a decision was made to replace the molar teeth in the clinical situation presented earlier, the most commonly recommended treatment options reported by the dentists were IFPDs (84.9%), metal frame RPDs (33%), acrylic RPDs (27.4%), and implants-retained RPDs (11.3%). Only 6.6% reported CFPD as an option.

### 3.3. Assessment of Dentists' Attitudes towards the SDA Concept and Its Application in Clinical Practice

Only those who were aware of the SDA concept were asked to fill this section. When asked whether the SDA concept could have a useful place in treatment planning in Jordan, 59 (67.8%) agreed, 10 (1.5%) did not agree, and 18 (20.7%) were not sure. Cost was reported as the main reason behind the answer by 54.2% of those who agreed. Reducing the number of visits was reported by 23.7%, and being more conservative was reported by 22%. The distribution of dentists according to the percentage of patients in which the SDA concept was applied is presented in Table 4.

## 4. Discussion

The current study was a cross-sectional survey of a random sample of dentists working in different work environments. The 70.7% response rate reported was in accordance with similar studies reported in the literature [12, 14, 17].

The majority of the dentists (82.1%) reported they were aware of the SDA concept. This percentage was compared favorably with the 61% awareness level reported by dentists in Australia [11] and was much higher than the 22.9% awareness level reported by dentists in India [16]. Dentists with fewer years of clinical experience were more likely to be aware of the SDA concept compared with dentists with more years of experience. They were also more likely to have learnt about this concept during their undergraduate education. Other surveys reported similar results where dentists with fewer years of experience were found to be more aware of the SDA concept [11, 16, 22]. This could be a reflection of the increased incorporation of the SDA concept in undergraduate teaching in the recent years [23].

When presented with the hypothetical clinical situation, 54.7% of the dentists did not agree that the molars should be replaced. Their decision was in accordance with evidence from the literature, which supports that patients could have good mastication ability without molar teeth [10, 24]. No association was found between awareness of the SDA concept and reporting no need for replacing the missing molars. The fact that many dentists were aware of the SDA concept did not necessarily indicate that they had thorough knowledge about the concept or that they would apply it in their decision-making for such a case. Similar results were reported by a study of Sudanese dentists [22]. Interestingly, some of the dentists who were unaware of the concept reported no need to replace the missing molars. This was in accordance with other studies from nighbouring Arab countires, which reported a decrease in dentists' inclination to replace missing posterior teeth as the age of the patient increased and the number of posterior teeth lost decreased [25, 26].

Improving mastication, restoring posterior support, preventing anterior tooth wear and maintaining the health of the TMJs were the main concerns for deciding to replace the missing molars. Similar concerns about the SDA concept were reported in other surveys [11, 23]. Satisfying patients' desires was also reported as a reason for providing treatment. This finding was similar to a study in Japan where they found that the patients preferred replacement of the missing molar teeth over no replacement [27].

On the other hand, participants who reported no need for replacing the missing molars stated that there was no functional or esthetic benefit from replacing those teeth and that it reduced the cost on the patients and simplified oral hygiene for them. Studies have indicated no significant improvement in masticatory function in patients with RPDs compared with those with premolar occlusion [28–31]. Cost was reported as a factor for selecting the SDA concept as a treatment modality by dentists in Saudi Arabia [18].

The most commonly recommended treatment modalities for replacing missing molar teeth were IFPDs (84.9%), followed by RPDs (33%; metal frame, 27.4%; acrylic RPDs). These results were in agreement with a study conducted in Saudi Arabia, where the IFPDs were the most preferred restoration option for the SDA by the majority of participating dentists, followed by metal frame RPDs, acrylic RPDs, and CFPDs [32]. On the other hand, these results were different from a study in the UK where IFPDs were found to be unpopular, and RPDs were the most popular among dentists [33]. Evidence from the literature reported poor compliance from patients with RPDs with distal extensions [30, 34]. However, other studies have indicated treatment with RPDs was preferred over no treatment from the patients' perspectives [35, 36]. Implant-retained RPDs were only reported by 11.3% of the dentists. This could be because most dentists and patients would opt for a fixed prosthesis over a removable one, if a decision to use implants was made. CFPDs were the least reported treatment option. It is worth mentioning that replacing missing molar teeth with IFPDs would add to the cost and the burden of maintenance [9].

A positive attitude towards the SDA concept was reported by 67.8% of the dentists. This percentage although high, was slightly lower than what was reported in other studies [11, 13–15]. The fact that the SDA concept is cost effective was the main reason behind believing the SDA concept could have a useful place in treatment planning in Jordan.

Of those who reported they were aware of the SDA concept, quarter of them did not apply it for any of their patients, and half of them only applied the SDA concept in <10% of their patients. It seemed that despite the high awareness percentage and the positive attitude towards the SDA concept, the majority of the dentists were not applying it in their clinical practice in Jordan. This finding was in agreement with similar studies conducted in other countries [13–15, 37, 38].

The SDA concept constitute a cost-effective approach to dental treatment with reduced burden of maintenance. However, despite these advantages, especially in developing countries such as Jordan, it is not commonly practiced as shown in the current study. Therefore, recommendations should be made to increase the awareness and knowledge about the SDA concept to encourge its application in clinical practice. Similar recommendations were made in other studies [13, 22].

This study assessed the attitudes and application of the SDA concept from the dentists' perspective. Assessing the SDA concept from patients' perspective is being currently conducted in Jordan. This is important since a discrepancy was found in the literature between the attitudes of dentists and patients towards the SDA concept. Moreover, the SDA concept does not seem applicable in all populations [39].

A limitation of the current study was that the presented clinical situation was for a patient aged 50 , with missing molar teeth and with favorable clinical conditions for accepting a SDA. Future research with more variable clinical scenarios should be conducted to further explore dentists' attitudes and decision-making regarding the SDA concept. Another limitation was that the clinical scenario presented took into account neither the patient's esthetic or functional concerns nor the patient's financial status. Such important factors always affect the decision-making process of the dentists in a real clinical setting.

## 5. Conclusion

The majority of the dentists in the current study were aware of the SDA concept and had a positive attitude towards it. However, most of the dentists did not apply it in their clinical practice. Cost reduction was an important reason behind the positive attitude towards the SDA concept. Those with fewer years of experience were more aware of the SDA concept.

## Figures and Tables

**Table 1:** The distribution of the sample according to gender, work environment, and years of clinical experience.

		Dentists	
		No.	%
Gender	Males	51	48.1
	Females	55	51.9

Work environment	Private practice	32	30.2
	University hospital	32	30.2
	Governmental institute	29	27.4

Level of education	General dental practitioners/interns	44	41.5
	Residents	35	33
	Specialists	27	25.5

Clinical experience	1-2 years	26	24.5
	3–5 years	29	27.4
	5–10 years	27	25.5
	>10 years	24	22.6

**Table 2:** Distribution of dentists who were aware/unaware of the SDA concept and where they learned about it (undergraduate/postgraduate), according to the years of clinical experience.

Years of clinical experience	Aware of SDA *N*. (%)	Not aware of SDA *N*. (%)	Undergraduate *N*. (%)	Postgraduate *N*. (%)
1-2	21 (24.1%)	5 (26.3%)	18 (36.7%)	4 (9.8%)
3–5	26 (29.9%)	3 (15.8%)	18 (36.7%)	9 (22.0%)
5–10	25 (28.7%)	2 (10.5%)	10 (20.4%)	15 (36.6%)
>10	15 (17.2%)	9 (47.4%)	3 (6.1%)	13 (31.7%)
Total	87 (100%)	19 (100%)	49 (100%)	41 (100%)
*X * ^2^ test	*X * ^2^ (3, *N*: 106) = 9.444, *P*=0.024^*∗*^		*X * ^2^ (3, *N*: 90) = 18.595, *P* < 0.001^*∗*^	

^*∗*^Statistically significant.

**Table 3:** Distribution of dentists who agreed/disagreed with the need of molar teeth replacement in the hypothetical case according to the awareness of the SDA concept.

Awareness of SDA concept	Agree *N* (%)	Do not agree *N* (%)
Aware	36 (75%)	51 (87.9%)
Not aware	12 (25.0%)	7 (12.1%)
Total	84 (100%)	58 (100%)
*X * ^2^ test	*X * ^2^ (1, *N*: 106) = 2.985, *P*=0.084^*∗*^	

**Table 4:** Distribution of the dentists according to the percentage of patients in which the SDA concept was applied.

Percentage of application of SDA concept	*N* (%)
Did not apply it	23 (26.4%)
<10%	44 (50.6%)
10–25%	14 (16.1%)
>25%	6 (6.9%)

## Data Availability

The SPSS data file used to support the findings of this study are available from the corresponding author upon request.
